# ^177^Lutetium-Dotatate delays decline in quality of life in patients with midgut neuroendocrine tumors

**DOI:** 10.18632/oncotarget.25942

**Published:** 2018-09-04

**Authors:** Jonathan Strosberg

**Affiliations:** Jonathan Strosberg: Department of GI Oncology, H. Lee Moffitt Cancer Center, Tampa, FL, USA

**Keywords:** ^177^Lutetium-Dotatate, NETTER-1, quality of life

Health-related quality of life (HRQoL) is increasingly viewed as an important and clinically relevant endpoint in oncologic clinical trials. Randomized studies offer the opportunity to evaluate the real-life impact of an investigational drug using time-to-deterioration (TTD) in HRQoL as an endpoint [1]. By generating a Kaplan Meier TTD curve with log-rank statistical comparison between the two randomized study groups, investigators can compare HRQoL decline in a manner analogous to comparison of radiographic time to tumor progression (TTP).

This methodology was recently used to analyze HRQoL of patients with advanced midgut neuroendocrine tumors (NETs) enrolled on the NETTER-1 study, a prospective phase III trial of ^177^Lutetium(Lu)-Dotatate versus high dose octreotide [2]. ^177^Lu-Dotatate is a novel radiolabeled somatostatin analog (SSA) designed to target somatostatin-receptor expressing NETs. Patients enrolled on the NETTER-1 study had biopsy proven well-differentiated midgut NETs and evidence of disease progression at baseline while on standard dose octreotide LAR (20mg or 30mg) [3]. During the study period, patients completed the European Organisation for Research and Treatment of Cancer (EORTC) quality-of-life questionnaires (QLQ) C-30 and G.I. NET 21. The former is a general cancer HRQoL questionnaire, and the latter is specific to gastrointestinal NET symptoms not addressed in the general questionnaire. These surveys were completed at baseline and every 12 weeks until disease progression. Per EORTC instructions, the results were converted to a 100 point scale. A decline of ≥10 points from baseline for an individual was considered clinically significant [4].

HRQoL questions can be categorized as “function scale” domains such as global health (overall QoL), physical functioning (activities of daily living), role functioning (advanced activities of daily living), and “symptom” domains such as pain, fatigue, dyspnea, appetite loss, diarrhea, etc. The function scale domains are clinically relevant for most cancer patients, whereas the relevance of individual symptoms can vary based on the cancer type. For example, diarrhea and flushing are particularly relevant to midgut NET patients, many of whom have carcinoid syndrome.

The HRQoL analyses represented a key secondary endpoint on the NETTER-1 study. Results showed a clinically and statistically significant improvement in TTD of global heath (hazard ratio [HR 0.41]), physical functioning (HR 0.52), and role functioning (HR 0.58) with ^177^Lu-Dotatate compared to high-dose octreotide. With respect to symptoms, there was significant improvement in TTD of diarrhea (HR 0.47), pain (HR 0.57), fatigue (HR 0.62) among other symptoms. There were no domains where improvement in TTD was seen with in the control arm [2].

These results provided an important validation that ^177^Lu-Dotatate not only improves progression-free survival (PFS) but also delays decline in quality of life and progression of symptoms. In the future, more randomized clinical trials should employ this emerging methodology to assess the impact of the investigational treatment on HRQoL. Moreover, standardized rules analogous to Response Evaluation Criteria in Solid Tumors (RECIST) should be developed to allow for uniform interpretation of HRQoL data across oncologic clinical trials.
